# Massive pulmonary tuberculosis cavity misdiagnosed as pneumothorax

**DOI:** 10.1002/rcr2.15

**Published:** 2013-09-06

**Authors:** John Kit-Chung Tam, Kee-Siang Lim

**Affiliations:** Department of Surgery, Yong Loo Lin School of Medicine, National University of SingaporeSingapore

**Keywords:** Bronchopleural fistula, pneumothorax, pulmonary cavity, reactivation tuberculosis, thoracostomy

## Abstract

Massive pulmonary cavity is a rare and serious complication of chronic reactivation tuberculosis. A 38-year-old gentleman had a history of tuberculosis treatment noncompliance 2 years ago. His presenting symptoms were cough, fever, and left-sided pleuritic chest discomfort for 2 months. Chest radiographs showed extensive lung destruction associated with large thick-walled cavities and severe fibrosis of the residual lung. In the emergency department, this was initially misdiagnosed as a large pneumothorax and a chest tube was inserted. Subsequently, this was misdiagnosed again as bronchopleural fistula when brisk air leak was seen. The chest tube did not lead to any radiological or clinical improvement and was removed without incident. This case demonstrates that massive pulmonary cavity can easily be misdiagnosed and tube thoracostomy is unnecessary. Although this condition was previously reported to be associated with a high mortality rate, our patient survived as a result of accurate diagnosis and prompt antituberculosis therapy.

## Introduction

Massive pulmonary cavity is a life-threatening complication of chronic reactivation tuberculosis (TB). There is a paucity of reports in the literature on this uncommon condition [1,2]. We present the report of a case of massive pulmonary cavity that was initially misdiagnosed as pneumothorax and subsequently misdiagnosed as bronchopleural fistula after chest tube insertion.

## Case Report

A 38-year-old gentleman presented to the emergency department with a 2-month history of cough, fever, and left-sided pleuritic chest discomfort. He had a past medical history of pulmonary TB diagnosed 2 years ago and was noncompliant with the prescribed anti-TB therapy. He had used intravenous drugs and had contact with commercial sex workers in the past. He had greater than 20 pack years of smoking history. There was no recent travelling history and no exposure to occupational or agricultural lung diseases.

On physical examination, the patient's temperature was 38.5°C. He was tachycardic (120 beats/min) and tachypneic (23 breaths/min). His oxygen saturation was 90% on room air, which improved to 99% with supplementary oxygen (4 L/min). Auscultation revealed markedly reduced air entry into the left lung.

**Figure 1:**
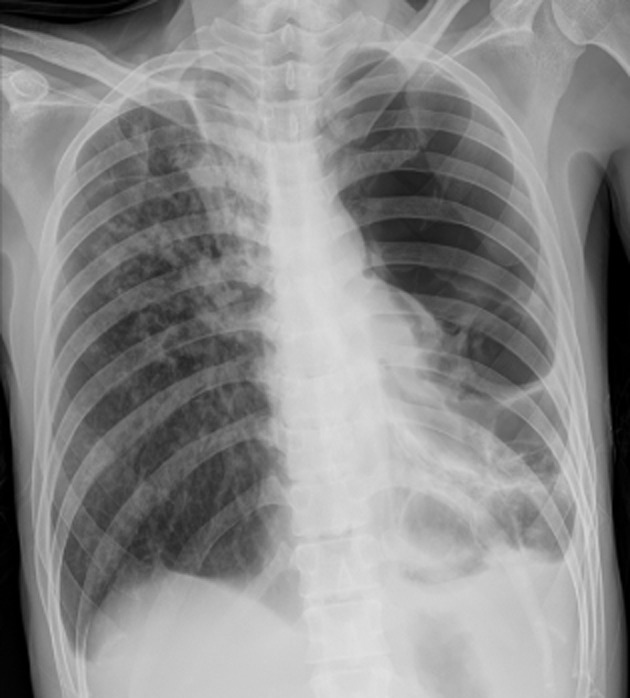
Chest radiograph showing massive pulmonary cavities in the left lung. Extensive areas of lucency in the left lung were present, resulting in the misdiagnosis of pneumothorax and unnecessary chest tube insertion. Patchy opacities and nodularities can be visualized on the right lung field.

**Figure 2:**
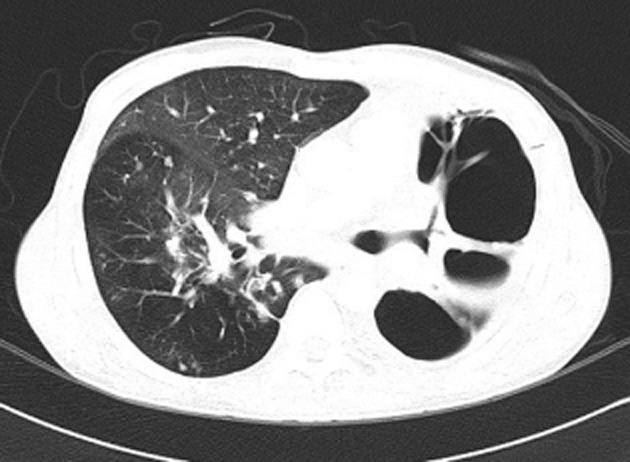
Computed tomography scan of the chest showed extensive lung destruction with multiple large thick-walled pulmonary cavities and severe fibrosis of the residual left lung. Diffuse fibro-nodular opacities were present in the right lung.

Blood test showed significant leukocytosis (21,000 white cells/μL) and highly elevated C-reactive protein (260 mg/L). Chest radiograph (Fig. 1) and computed tomographic scan of the chest (Fig. 2) showed massive pulmonary cavities in the left lung, as well as multiple nodular opacities in the contralateral right lung.

The patient was initially misdiagnosed by the emergency room physicians as having a large left pneumothorax and a chest tube was inserted. Brisk air leak was observed from the chest tube, and the patient was subsequently misdiagnosed as having a bronchopleural fistula. No improvement of the left lung was seen radiographically after chest tube placement, and it was later removed without complications.

Sputum TB molecular testing using the GeneXpert polymerase chain reaction (PCR) test was positive in two sputum samples. RpoB mutation was not detected in both samples. A total of five sputum samples were sent for acid fast bacilli smear and TB culture and were negative. Sputum culture also grew light *Pseudomonas aeruginosa* and light yeast. Based on the TB PCR result and the characteristic radiographic appearance on computed tomography (CT), the diagnosis was confirmed to be massive pulmonary cavity with extensive left lung destruction due to chronic TB reactivation and bacterial superinfection. He was admitted under isolation and was started on a multidrug TB regimen comprising of rifampicin, isoniazid, ethambutol, and pyrazinamide administered under directly observed therapy. Antibiotic was also administered to treat the isolated *P. aeruginosa*. The patient was eventually discharged from the hospital after 2 weeks, and his treatment was continued in the outpatient Tuberculosis Care Unit.

## Discussion

The global disease burden and societal cost of TB are high. According to the World Health Organization Global Tuberculosis Control 2010, the estimated global TB incidence was 9.4 million cases whereas the estimated global TB prevalence was 14 million cases. Reactivation of primary pulmonary TB is a challenging medical problem. Several risk factors are associated with TB reactivation, including age, malnutrition, human immunodeficiency virus infection, malignancy, immunosuppressive drugs, and incomplete or inadequate treatment for active pulmonary TB. These risks factors disrupt the balance between host and pathogen. Reactivation of pulmonary TB is due to containment failure of dormant *Mycobacterium tuberculosis* inside granulomas. *M. tuberculosis* is reactivated through up-regulation of resuscitation-promoting factors and matrix metalloproteinase-1, which results in destruction of the extracellular matrix of the lung [3,4]. Decaying and rupturing of the granulomas result in liquefaction, caseation, and cavitation of the lung. Intense inflammation may result in widespread arteritis and vascular thrombosis, with resulting pulmonary ischemia, necrosis, and gangrene [1]. Parenchymal destruction can occasionally be worsened by lymph node obstruction of the bronchi with distal collapse [5].

Patients typically present with symptoms of fever, weight loss, night sweats, progressive dyspnea, cough, hemoptysis, and pleuritic chest pain. Large thick-walled cavities, severe fibrosis of the residual lung, and occasionally air-fluid levels may be seen radiographically [5].

This case report illustrates that patients with massive pulmonary cavities and extensive lung destruction may initially be misdiagnosed on chest X-ray as having a large pneumothorax, severe emphysematous lung disease, giant bullae, or bronchopleural fistula. If no fluid is present within the cavities and no empyema is present, chest tube drainage is unnecessary and does not aid in clinical or radiographical improvement.

Although most patients in chronic TB reactivation will still be susceptible to standard TB treatment, the possibility of multidrug-resistant TB should be evaluated. Prompt anti-TB treatment is essential, and antibiotics or antifungal agents are needed if bacterial or mycotic superinfection is present. If medical treatment is unsuccessful, surgery may occasionally be considered to debride necrotic lung tissue, drain empyema, or manage complications such as broncho-pleural fistula.

Overall prognosis is poor and the diagnosis of pulmonary cavity carries a high mortality rate. In one small series published 30 years ago, three out of four (75%) patients with TB who developed pulmonary cavity died [1]. Since then, there is a paucity of published evidence on this condition. The patient in this report survived because of his younger age and prompt institution of anti-TB therapy.

In conclusion, massive pulmonary cavity secondary to reactivation TB is a rare and serious complication that can be misdiagnosed as pneumothorax and bronchopleural fistula. CT appearance is characteristic with large thick-walled cavities and severe fibrosis of the remaining lung parenchyma. This diagnosis should be suspected in patients who present with a history of TB and the characteristic radiographic appearances. Tube thoracostomy is unnecessary and should be avoided. Vigilance to establish accurate diagnosis is essential and prompt TB treatment can be life saving.
